# mTOR Regulation of Lymphoid Cells in Immunity to Pathogens

**DOI:** 10.3389/fimmu.2016.00180

**Published:** 2016-05-11

**Authors:** Rachael Keating, Maureen Ann McGargill

**Affiliations:** ^1^Department of Immunology, St. Jude Children’s Research Hospital, Memphis, TN, USA

**Keywords:** mTOR, immune regulation, pathogen, metabolism, immune differentiation

## Abstract

Immunity to pathogens exists as a fine balance between promoting activation and expansion of effector cells, while simultaneously limiting normal and aberrant responses. These seemingly opposing functions are kept in check by immune regulators. The mechanistic target of rapamycin (mTOR) is a serine/threonine kinase that senses nutrient availability and, in turn, regulates cell metabolism, growth, and survival accordingly. mTOR plays a pivotal role in facilitating immune defense against invading pathogens by regulating the differentiation, activation, and effector functions of lymphoid cells. Here, we focus on the emerging and sometimes contradictory roles of mTOR in orchestrating lymphoid cell-mediated host immune responses to pathogens. A thorough understanding of how mTOR impacts lymphoid cells in pathogen defense will provide the necessary base for developing therapeutic interventions for infectious diseases.

The mechanistic target of rapamycin (mTOR) is an evolutionarily conserved serine/threonine kinase that is ubiquitously expressed in immune cells. mTOR integrates multiple environmental signals to regulate diverse cellular processes including protein translation, cell growth, proliferation, metabolism, migration, and survival. Accordingly, mTOR plays a key role in multiple components of both myeloid and lymphoid cell differentiation, activation, and acquisition of effector functions. Through regulation of these key mechanisms, mTOR has an essential role in generating and regulating immune cells to combat pathogens.

The emerging view describes mTOR not as a single trigger for a linear cascade of events, but rather as a multifunctional orchestrator of diverse immune responses. In this review, we detail the current understanding of mTOR as a multifaceted regulator of immunity to pathogens through its impact on lymphoid cells. Specifically, we describe mTOR regulation of natural killer (NK) cells, invariant natural killer T (iNKT) cells, CD8 and CD4 T cells, and B cells during immunity to pathogens. The regulation of myeloid cells has been extensively reviewed recently (1). We also explore how mTOR inhibition may be utilized to enhance immunity to pathogens and discuss implications for vaccine design.

## mTOR Complexes and Signaling Cascades

**Figure 1:**
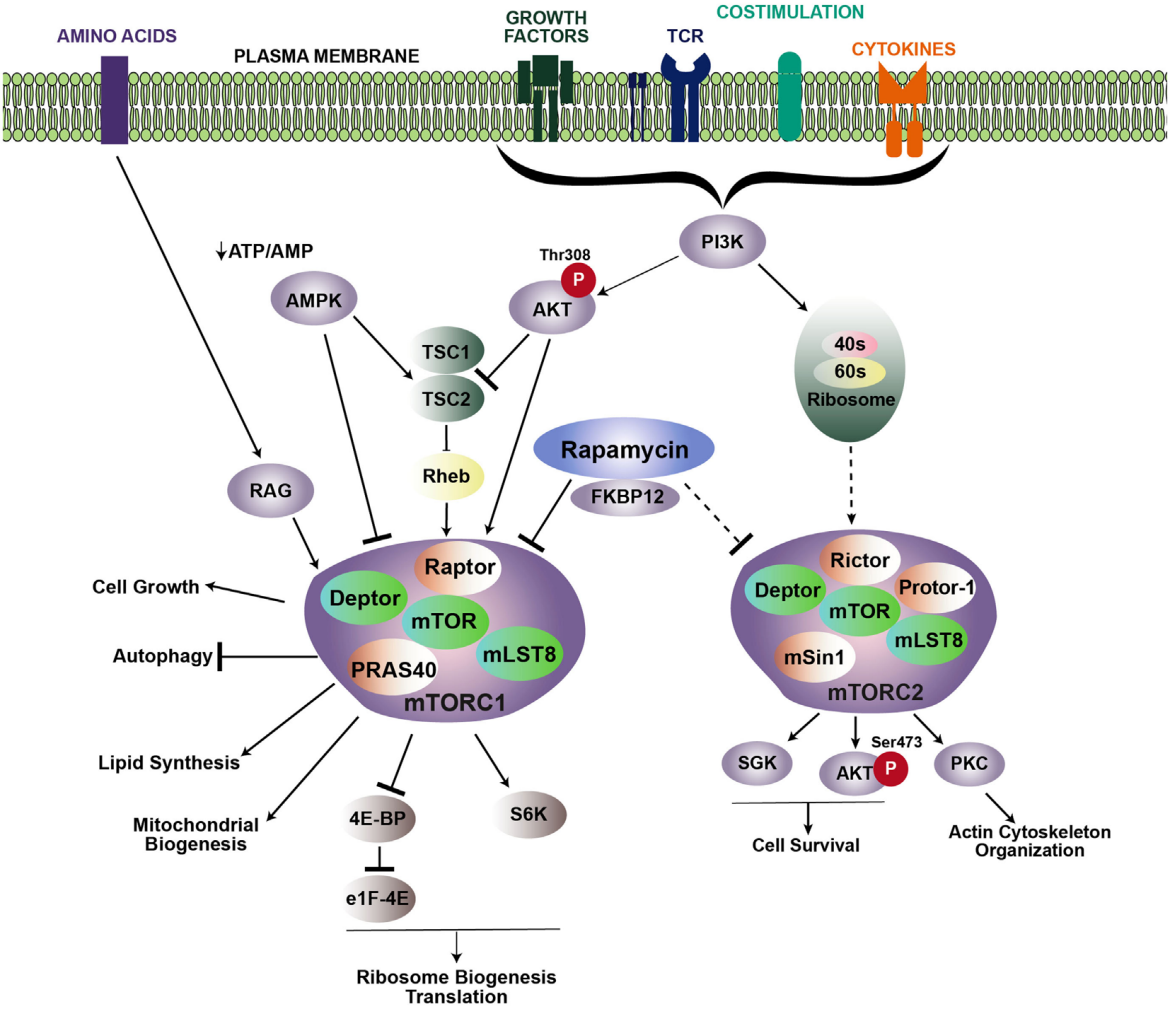
**Signaling cascades promoting mTOR activation**. mTOR regulates multiple cellular processes via two distinct complexes: mTORC1 and mTORC2. Growth factors, TCR engagement, costimulation, and cytokines all contribute to PI3K activation, which leads to the recruitment of Akt to the membrane, where it is phosphorylated at position threonine 308. Activated Akt then phosphorylates the TSC1/TSC2 inhibitory complex, which releases Rheb and induces accumulation of Rheb-GTP to promote mTORC1 activity. Rapamycin inhibits mTOR by binding to the intracellular partner, FKBP12, which directly inhibits mTORC1. Upstream regulation of mTORC2 is less known, but is downstream of PI3K activation. Association with ribosomes regulates mTORC2 activation. mTORC2 is typically regarded as insensitive to rapamycin; however, prolonged rapamycin treatment can reduce mTORC2 activity. Arrows and bars represent activation and inhibition, respectively. Dashed lines indicate that the exact mechanism is unknown.

Mechanistic target of rapamycin functions as two signaling complexes in mammalian cells: mTOR complex 1 (mTORC1) and mTOR complex 2 (mTORC2) (Figure 1). mTORC1 includes a scaffolding protein, regulatory-associated protein of mTOR (Raptor), DEP-containing mTOR interacting protein (Deptor), mammalian lethal with Sec13 protein (mLST8), and the Proline-Rich AKT substrate (PRAS40). Similarly, mTORC2 also comprises mTOR, Deptor, and mLST8, with the addition of the scaffold protein Raptor-independent Companion of TOR (Rictor), the Protein observed with Rictor (Proctor), and the mammalian stress activated protein kinase-interacting protein 1 (mSIN1).

mTOR complex 1 is activated in response to various extracellular stimuli including nutrients, growth factors, stress, cytokines, and antigen receptor signaling. When nutrients and these stimuli are readily available, mTORC1 activity is high, and energy-demanding cellular processes such as translation and ribosomal biogenesis are promoted. Extracellular stimuli activate mTORC1 by triggering a signaling cascade through PI3 kinase (PI3K) and the protein kinase, Akt. Akt then phosphorylates the mTORC1 repression factor, consisting of tuberous sclerosis complex 1 (TSC1) and TSC2. Phosphorylation of the TSC complex prevents it from inhibiting Rheb, which is essential for mTORC1 activation. Additionally, mTORC1 can also be activated in a TSC-independent process, whereby Akt blocks inhibition of mTORC1 by phosphorylating PRAS40 to stabilize the mTOR–Raptor interaction (2, 3). Activation of mTORC1 leads to phosphorylation of p70–S6 kinase (S6K) and the eukaryotic initiation factor 4E-binding protein (4E-BP1), which helps regulate translation (4–6). mTORC1 signaling also increases the rate of glycolysis by inducing the expression of HIF-1α and c-Myc. Conversely, mTORC1 activity is inhibited when nutrient-associated cues are lacking. For example, in response to a decrease in the ATP/ADP ratio, the AMP-activated protein kinase (AMPK) becomes activated, and in turn, inhibits mTOR activity by phosphorylating TSC2 or Raptor.

Activation of mTORC2 is not as well defined as mTORC1. Similar to mTORC1, activation of mTORC2 occurs downstream of P13K. However, activation of mTORC1 occurs downstream of Akt following phosphorylation at threonine 308 (T308), while mTORC2 acts upstream of Akt by phosphorylating serine 473 of Akt (S473). In contrast to mTORC1 activation, mTORC2 activation occurs independent of protein synthesis, but instead, relies on association with the ribosome (7). Activation of Akt by mTORC2 also leads to phosphorylation and inhibition of FOXO1 and FOXO3 (8). In addition to Akt, mTORC2 also activates serum glucocorticoid-regulated kinase 1 (SGK1) and protein kinase C alpha (PKCα). Signaling via mTORC2 is important for reorganization of the cytoskeleton through activation of RhoA GTPase and promoting cell survival by upregulating anti-apoptotic proteins (7, 9).

Much of the role and function of mTOR has been ascertained with rapamycin, a small molecule drug derived from *Streptomyces hygroscopicus*. Rapamycin binds a 12-kDa Fk506-binding protein (FKBP12) to form the rapmycin–FKBP12 complex, which binds mTOR and inhibits formation of the mTORC1 complex (10). Prolonged rapamycin exposure also inhibits mTORC2 (11). However, mTORC2 is typically regarded as insensitive to rapamycin. Use of knock-out mice, in particular, mice with a conditional deletion of *Raptor*, *Rictor*, *Rheb*, or *Tsc*, which are critical components of mTORC1 and mTORC2 complexes, have also greatly advanced our understanding of mTOR signaling pathways and function (Table 1).

**Table 1:** **Overview of studies demonstrating the role of mTOR in lymphoid cells following pathogen infection**.

Cell type	mTOR modification	Pathogen	Cellular and pathogen outcome	Conclusion about mTORs activity during infection	Reference
NK cells	Rapamycin	MCMV	Blocked proliferation, IFN-γ synthesis and granzyme B expression. Higher viral titer	Promotes proliferation, IFN-γ synthesis and granzyme B expression, and pathogen clearance	(33)
	*mTOR*^−/−^ NK cells	MCMV	Blocked proliferation and granzyme B expression. Higher viral titer	Promotes proliferation and granzyme B expression and pathogen clearance	(31)
CD8 effector	T cell-specific *Tsc2* deletion to enhance mTORC1 activity	Vaccinia-OVA	Excessive generation of effector CD8^+^ T cells, unable to differentiate into memory cells. High cytolytic activity. Robust IFN-γ and TNF-α	mTORC1 promotes generation of effectors and mTORC1 suppression promotes memory formation	(57)
	T cell-specific *Rictor* deletion to inhibit mTORC2	Vaccinia-OVA	Unaltered CD8 differentiation and effector function	mTORC2 does not regulate effector cells	(57)
	T cell-specific *Rheb* deletion to inhibit mTORC1	Vaccinia-OVA	Reduced CD8 effector function. Decreased IFN-γ, TNF-α, and cytolytic function	mTORC1 enhances CD8 effector function	(57)
	Rapamycin	LCMV and *Listeria*	Impaired CD8 effector function and reduced pathogen clearance	mTORC1 promotes effector function and pathogen clearance	(59)
	Rapamycin	Influenza	Reduces IRF4 expression, which is required for effector CD8 T cell differentiation and expansion. Impaired viral clearance and host recovery	mTOR regulates IRF4 expression to impact during CD8 T cell differentiation to promote pathogen clearance	(61)
	Rapamycin	LCMV and LM-OVA	Impaired effector CD8 T cell number and function, Reduced IFN-γ, TNF-α, granzyme B, and cytolytic activity. Reduced pathogen clearance and survival	mTORC1 promotes effector function, pathogen clearance, and host survival	(59)
CD8 memory	Rapamycin, *mTOR*, and *Raptor* deletion	LCMV	Enhanced memory cell quantity, quality, and persistence	mTORC1 suppresses memory quality and quantity	(67)
	*In vitro* rapamycin treatment prior to cell transfer	LCMV-gp 33 peptide	Enhanced and long-lived memory cell formation	mTORC1 suppresses memory formation	(69)
	*In vitro* rapamycin treatment of WT and *TRAF6*^−/−^ cells prior to cell transfer and infection	LM-OVA	Restored the ability to develop memory cells and increased the recall response in the absence of *TRAF6*	mTOR blocks memory development and recall responses	(68)
	T cell-specific *Tsc2* deletion to enhance mTORC1 activity	LM-OVA	Effector cells were unaltered. Differentiation of effector cells to memory cells was impaired. Recall response was reduced	Excessive mTORC1 activity inhibits memory formation and is regulated by Tsc1	(70)
	Rapamycin	LCMV and LM-OVA	Enhanced CD8 memory formation	mTOR suppress memory formation	(59)
	Rapamycin	Canary poxvirus	Long-term, low dose rapamycin blocked memory formation. Short-term, high dose rapamycin enhanced CD8 memory	Sustained, low level mTOR activity supports memory formation	(71)
	Rapamycin	Vaccinia virus	IL-12-dependent increase in memory CD8 T cells	IL-12 regulates the mTORC1 block in formation of memory CD8 T cells	(76)
	T cell-specific *Rictor* deletion to inhibit mTORC2	Vaccinia-OVA	Enhanced generation of memory CD8 T cells	mTORC2 limits memory cell formation	(57)
CD8-resident memory	Rapmycin shRNA silenced mTOR	Vesicular stomatitis virus (VSV) and VSV-OVA	Rapamycin increased the quantity of memory CD8 in the spleen but reduced resident memory cells in the intestinal mucosa and vaginal mucosa	mTOR enhanced formation of memory cells in the intestinal and vaginal mucosa	(84)
CD8 secondary expansion	Rapamycin	LCMV, Pichinde virus	IL-15-dependent, virus-induced cell cycling of memory CD8 cells was blocked	Inflammatory IL-15 activates the mTORC1-signaling pathway to support preexisting memory cells and enhance antiviral protection	(78)
CD8 T cell exhaustion	Rapamycin	Chronic LCMV	Abrogated therapeutic effects of blocking PD-1, leading to CD8 T cell exhaustion and failure to control chronic infection	During chronic infection persistent antigen impairs mTOR activation, allowing FOXO1 activity to increase and promote differentiation of terminally exhausted CTLs	(79)
Tfh cells	shRNA silenced *Rictor* or *Raptor*	LCMV	Raptor silencing favored Tfh development over Th1 development. Rictor silencing favored Th1 over Tfh development	IL-2-mediated mTORC1 activation promotes Th1 over Tfh development. mTORC2 activation favors Tfh over TH1 development	(118)
B cells	Mice hypomorphic for *mTOR* and B cell-specific deletion of *mTOR*	*S. pneumoniae*	Decreased germinal centers, high-affinity antibodies, and SMH/CSR. Higher mortality in hypomorphic mTOR mice	mTOR is a critical immunoregulator, promoting germinal center formation through AID signaling to generate high affinity antibodies	(125)
	ATP-competitive mTOR kinase inhibitor (TOR-KIs)	*Salmonella*	Early (d14) IgM response was unaltered and IgG2c decreased. Late (d28) IgM increased and Tfh cell% increased with some evidence of increased GC B cells	Partial inhibition of mTOR activity increases protective IgM responses	(138)
	Rapamycin	Influenza vaccination and heterosubtypic challenge	Delayed germinal center formation, reduced class switching, increased survival. Increased viral clearance	mTOR supports antibody class switching and affinity maturation, which may impair viral clearance to heterosubtypic infection	(137)

Several pathways regulate activation of mTORC1 and mTORC2. When energy levels are low, mTORC1 is inactivated, and FOXO transcribes *Rictor*, promoting formation of mTORC2, which phosphorylates Akt S473 (12). Transcription of *Rictor* and subsequent phosphorylation of Akt S473 requires mTORC1 inhibition (13). Similarly, while mTORC1 activates protein synthesis and S6K, S6K activity can repress Rictor and mTORC2 function. In addition, recent studies highlight a positive feedback loop between Akt and mTORC2 via SIN1 phosphorylation, whereby Akt is activated following PDK1 phosphorylation. Next, Akt phosphorylates SIN1, enhancing mTORC2 activity, which then promotes phosphorylation and complete activation of Akt (14).

Pathogens can also influence activation of the mTOR pathway. mTORC1 regulates translation by phosphorylating 4E-BP1, which releases it from the 5′ cap-binding protein, eukaryotic translation initiation factor 4E (eIF4E) allowing translation to proceed (4–6). Pathogens that are dependent on the host’s cellular 5′ cap-dependent translation must therefore maintain mTOR activity, or bypass the need for mTOR-mediated phosphorylation of 4E-BP1 to enable the translation complex to form. Indicative of the former approach, human papillomavirus (HPV) uses two early proteins, E6 and E7, to activate mTOR signaling, which phosphorylates and inactivates 4E-BP1 to support viral cap-dependent protein synthesis (15, 16). Similarly, Epstein–Barr virus (EBV) activates cap-dependent translation using a viral protein, LMP2A, to activate mTORC1 (17). Adenovirus also uses viral proteins (e4-ORF1 and e4-ORF4) to mimic stimulatory signals and activate mTORC1 activity in the absence of nutrients or growth factors to maintain translation of viral proteins (18). Bacterial pathogens including *Listeria monocytogenes* (*L. monocytogenes*) and *Staphylococcus aureas* can also activate mTOR to promote IL-10 production and increase their survival in the host (19). Alternatively, some pathogens have evolved mechanisms to bypass mTORC1 activity. For example, human cytomegalovirus (HCMV) bypasses mTORC1 activity by directly phosphorylating 4E-BP1 and eIF4G to maintain the activity of the translation complex (20). In contrast, some pathogens such as *Leishmania major* have proteases that block mTOR activation, which suppresses the type 1 IFN response, allowing the pathogen to survive within cells (21). Hence, a pathogen’s translation requirements and the ability to resolve these requirements will influence whether the pathogen tries to enhance, bypass, or suppress mTOR activity, and in turn, will influence the counter approach by the host immune response.

## mTOR Regulation of Autophagy in Host Defense

Mechanistic target of rapamycin regulates cell processes in response to nutrient availability. A key component of cellular control by mTOR is through regulation of autophagy, which is an essential process in all myeloid and lymphoid cells. Autophagy facilitates turnover of unnecessary or damaged cellular components. These cellular components are surrounded by a double-membraned vesicle, targeted to a lysome, degraded, and then recycled. This process allows cells to survive under stress. When energy sources are low, mTOR activity is low, biosynthesis is attenuated, and autophagy is upregulated to recycle nutrients, rather than synthesize new material. This prevents translational arrest and cell death. Conversely, when energy and nutrients are readily available, mTOR is active and signals downstream pathways to generate new cellular material to promote cell growth and proliferation, while suppressing autophagy. Basal autophagy levels are essential for homeostatic clearance of protein aggregates and damaged organelles (22). Basal autophagy is regulated independent of mTOR; however, mTOR suppresses autophagy induction above basal levels (23). Regulation of autophagy by mTOR provides an interface for both pathogen assault and host defense, as intracellular pathogens compete with the host for energy and resources.

Stimuli triggered by pathogen infection can induce autophagy above basal levels to destroy intracellular pathogens, while simultaneously increasing the cell surface presentation of microbial antigens to stimulate the immune response. For example, infection with the bacteria, *Shigella flexneri*, causes amino acid starvation and subsequent downregulation of mTOR to induce autophagy (24). The adaptation of the immune system to detect and respond to intracellular pathogens has simultaneously provoked evolution of some pathogens to circumvent autophagy induction. Indeed, HSV-1 and HSV-2 prevent induction of autophagy to evade immune defense mechanisms (22, 25, 26). Similarly, *L. monocytogenes* and *Salmonella* attempt to subvert induction of autophagy by reactivating mTOR to downregulate the immune response (24, 27). Therefore, these pathogens hijack and maintain basal levels of autophagy to exploit host energy supplies and nutrients for their own replication. In such situations, it is beneficial for host defense mechanisms to inhibit mTOR and induce autophagy above basal levels.

In contrast, autophagy induction can benefit some pathogens by supporting their replication. Multiple subtypes of influenza A virus induce autophagy and autophagic cell death by suppressing mTOR to promote replication (28, 29). Hence, autophagy inhibitors may limit influenza virus infection. Datan et al. also reported that influenza virus infection induced autophagy in apoptotic cells in the presence of mTORC1 and mTORC2 activity, indicating that alternate regulatory mechanisms may override suppression of autophagy by mTOR (30). The degree to which various pathogens support or inhibit mTOR activity is therefore a reflection of the extent to which autophagy benefits or hinders their own replication, and the degree to which they have evolved to counter host immune responses.

## mTOR in NK Cell Activation and Effector Functions

Natural killer cells are a subset of innate lymphoid cells that limit infection by intracellular pathogens and promote tumor immunosurveillance. The name “natural killer” reflects their capacity to kill target cells without prior antigenic stimulation. NK cells develop from common lymphoid progenitor cells in the bone marrow in a process dependent on proliferation and mTOR. Mice with a conditional deletion of *mTOR* in NK cells had a dramatic reduction in NK cell development and differentiation due to defects in proliferation (31). Similarly, transplant patients treated with rapamycin had reduced NK cell numbers (32).

Following development and maturation in the bone marrow, NK cells enter the periphery in a metabolically resting, quiescent state. In the periphery, exposure to IL-15 or viruses promotes NK cell activation, leading to an increase in metabolism, cytokine production, and acquisition of cytotoxic effector functions. High IL-15 concentrations are required to activate mTOR (31). Complete NK cell activation requires mTOR signaling, as rapamycin and NK cell-specific deletion of *mTOR* blocked proliferation and granzyme B expression in response to *in vitro* cytokine stimulation, *in vitro* polyI:C stimulation, and MCMV infection (31, 33, 34). Consequently, inhibition of mTOR and NK function resulted in higher viral titers following MCMV infection (31, 33). Interestingly, IFN-γ secretion was unimpaired following MCMV infection in mice with a NK cell-specific mTOR deletion, yet it was blocked in MCMV-infected mice treated with rapamycin (33). This difference could be due to the fact that rapamycin inhibits mTOR in cells other than NK cells, suggesting that mTOR signaling in other cell types may impact NK cell effector functions. Alternatively, rapamycin may inhibit the mTOR pathway to a different extent than genetic deletion of mTOR in NK cells.

mTOR complex 1 regulates NK cell effector function by enhancing glucose uptake and promoting aerobic glycolysis. Accordingly, directly limiting glycolysis inhibits IFN-γ production and granzyme B expression by NK cells (35). Cells infected with pathogens typically increase glucose uptake and glycolysis and therefore, limit the amount of glucose available to surrounding immune cells (36). The effector functions of NK cells may therefore be hindered by the availability of glucose following infection. During the initial phase of MCMV infection, proliferation of NK cells is IL-15 and mTOR dependent. However, NK cell proliferation subsequently becomes IL-15 and mTOR independent, at which point proliferation is driven by activating receptors on the NK cell, such as Ly49H, which recognizes viral ligands on infected cells, but does not activate mTOR (37, 38). The later, mTOR-independent phase of such immune responses may be an adaptation to maintain NK effector functions with diminishing glucose supplies. Regardless, this model illustrates that immune response kinetics influence the requirement for mTOR signaling in pathogen defense.

## mTOR in iNKT Cell Development, Activation, and Effector Functions

Signaling through mTOR is also important in the development, activation, and effector function of invariant NKT cells (iNKT) cells. iNKT cells share features common with both NK cells and T cells. Similar to NK cells, iNKT cells rapidly produce large amounts of cytokines following activation, including IFN-γ, TNF-α, IL-4, and IL-17. However, unlike NK cells, iNKT cells express a T cell receptor (TCR) similar to conventional T cells, albeit at intermediate levels and with decreased diversity. In mice, the iNKT TCR consists of a Vα14Jα18 α-chain paired with Vβ2, Vβ7, or Vβ8.2 β chains. The iNKT cell TCR recognizes lipid antigens bound to the non-classical major histocompatibility complex (MHC) homolog, CD1d. These cells contribute to tumor immunity, autoimmunity, and immunity to pathogens.

Invariant natural killer T cells can be divided into subsets based on transcription factor expression and cytokine production. Expression of the transcription factors T-bet, GATA3, and RORγt define the NKT1, NKT2, and NKT17 subsets, respectively (39, 40). Mice with a conditional deletion of *Raptor* in T cells showed that mTORC1 is required for the development of the NKT1 subset, and to a lesser degree, NKT2 cells (41, 42). Furthermore, the remaining iNKT cells were functionally compromised, as they failed to produce IFN-γ and TNF-α following stimulation with α-Gal-Cer. These data indicate that mTORC1 is differentially required for NKT cell development and effector functions.

While mTORC1 was required for development of NKT1 and NKT2 cells, mice with abrogated mTORC2 signaling had a defect in NKT17 development (43). Moreover, development of NKT17 cells was enhanced in a *Rictor*-dependent manner in the absence of PTEN, further clarifying that differentiation of the NKT17 lineage is mTORC2 dependent, but mTORC1 independent. However, Prevot et al. found that mTORC2 was required for the development of NKT2, but not NKT1 or NKT17 cells (44). The reason for this inconsistency is not known as both studies used mice with a conditional deletion of *Rictor* in T cells for their studies. Possible factors that contribute to such variable findings include diverse microbiomes associated with the different facilities, use of different markers to identify iNKT cell subsets, and technical difficulties associated with the reagents available to analyze iNKT cell transcription factors. An intriguing study recently showed that in the presence of IL-10 and rapamycin, iNKT cells expressed Foxp3 and acquired properties associated with immunosuppressive regulatory cells, identifying yet another population of iNKT cells: Foxp3^+^ iNKT cells (iNKTregs) (45).

Stimulation of iNKT cells with α-Gal-Cer activates mTOR signaling (46). Mice with an inducible deletion of *Raptor* in mature iNKT cells showed that mTORC1 signaling is required for optimal proliferation and IL-4, IFN-γ, and TNF-α synthesis following *in vitro* and *in vivo* stimulation with α-Gal-Cer (42). Moreover, *Raptor* deficiency inhibited iNKT-mediated autoimmune hepatitis, which arises in part, due to TNF-α produced by iNKT cells, further highlighting a role for mTOR in regulating iNKT cell effector function.

A role for iNKT cells in pathogen defense is clearly evident following their activation with artificial ligands during infection with different pathogens including malaria (47), HIV (48), influenza virus (49), and hepatitis B virus associated hepatic carcinoma (50). In addition, iNKT cells mediate pathogen defense against several strains of bacteria that express microbial glycolipids and diacylglycerols capable of binding CD1d molecules, such as *Streptococcus pneumoniae* (51, 52), *Novosphingobium aromaticivorans* (53), and *Borrelia burgdorferi* (52). At present, mTOR has not been implicated in pathogen responses mediated by iNKT cells. However, as mTOR is required for the development and function of iNKT cells, it is perhaps only a matter of time before a role for mTOR in regulating pathogen defense by iNKT cells is established.

## mTOR in CD8 T Cell Immunity to Pathogens

CD8^+^ T cells play a pivotal role in host defense against pathogens by directly killing infected cells via release of cytotoxic granules containing granzymes and perforin, and indirectly through cytokine secretion. A critical role for mTOR is implicated in several stages of CD8 T cell-mediated immunity including activation, differentiation, migration, and memory formation.

Naive CD8^+^ T cells undergo sequential stages of activation, clonal expansion, and differentiation to generate pathogen-specific effector CD8^+^ T cells. Activation of naive CD8^+^ T cells requires recognition of their cognate antigen presented by MHC class I molecules on the surface of antigen-presenting cells (APC). Engagement of the TCR with antigen and MHC, in the context of costimulation, induces PI3K signaling and subsequent activation of mTORC1 via TSC1-dependent and -independent processes. Activation of mTORC1 independent of TSC1 occurs through binding of the chaperone protein, Hsp90 to Raptor. The interaction of Hsp90 and Raptor promotes mTORC1 activation and prevents T cell anergy, as activation of T cells during a blockage of Hsp90 led to T cell tolerance (54).

One way in which mTOR regulates T cell activation is by blocking negative regulators of T cell activation. In particular, naive CD8^+^ T cells are kept in a state of quiescence by several transcription factors including KLF4 and E74-like factor 4 (ELF4). Signaling through mTOR inhibits KLF4 and ELF4 and reverses quiescence following TCR engagement, allowing CD8^+^ T cell activation and proliferation to proceed (55).

Following CD8^+^ T cell activation, antigen-specific T cells undergo clonal expansion and differentiate into effector cells. Progression to effector CD8^+^ T cells is coordinated with a switch from catabolism to anabolism and oxidative glycolysis. CD8^+^ T cells drastically increase their glucose metabolism as they respond to pathogens and differentiate into effectors (56). Signaling through mTORC1 promotes glycolysis in effector CD8^+^ T cells, and mTOR inhibition with rapamycin suppresses this process. Indeed, CD8^+^ T cells deficient in mTORC1 signaling failed to develop into effector cells following Vaccinia infection (57, 58). Likewise, rapamycin treatment during crucial stages of infection impaired glycolysis and impeded CD8^+^ effector function and pathogen clearance during infection with lymphocytic choriomeningitis virus (LCMV) and *L. monocytogenes* (59). Conversely, genetic deletion of *Tsc2* and enhanced mTORC1 activity led to excessive generation of CD8^+^ effector cells. Importantly, constitutively activate mTORC1 produced terminally differentiated effector cells and impaired memory development (57). While mTORC1 activity is required to generate effective CD8^+^ effector cells, mTORC2 appears to be dispensable as mice with a conditional deletion of *Rictor* exhibit normal proliferation and effector functions (57).

Effector differentiation of CD8^+^ T cells is tightly regulated by several transcription factors. The transcription factor HIF-1α is required to sustain glucose metabolism and expression of perforin and granzymes in effector CD8^+^ T cells (58). HIF-1α controls glycolysis by regulating expression of the glucose transporter, Glut1. As CD8^+^ T cells differentiate into effectors, they increase expression of HIF-1α in an mTORC1- and IL-2-dependent manner. Therefore, inhibition of mTORC1 with rapamycin inhibits HIF-1α and Glut1 expression, glucose uptake, and glycolysis (58). Signaling through mTOR also promotes glycolysis via the oncogene c-MYC, which regulates metabolic reprograming in T cells following activation (60). Expression of c-MYC is upregulated in an mTOR-dependent manner following TCR stimulation and influences the expression of rate-limiting glycolytic enzymes (60).

Although the HIF-1α complex is required to sustain glycolytic metabolism in CD8^+^ T cells, it is not required to initiate the process nor is it essential for T cell proliferation (58). In contrast, the transcription factor interferon regulatory factor 4 (IRF4) was critical for proliferation and survival of CD8^+^ T cells responding to infection with influenza virus and *L. monocytogenes* (61, 62). IRF4 maintains aerobic glycolysis and effector functions in activated CD8^+^ T cells by promoting the expression of multiple glycolytic enzymes (62). IRF4 expression in CD8^+^ T cells is regulated by mTOR, and is proportional to the strength of TCR stimulation, such that strong TCR stimulation increases mTOR activity, which enhances IRF4 expression and CD8^+^ T cell expansion and effector differentiation. Conversely, rapamycin treatment reduced mTOR signaling and impaired IRF4 expression and CD8^+^ T cell differentiation, leading to impaired viral clearance and host recovery from infection (61).

SEMA4A is a class IV semaphorin that activates mTORC1 signaling and is also required for optimal CD8^+^ T cell activation and differentiation (63). SEMA4A-mediated mTOR signaling was not important in the early phases of CD8^+^ T cell activation, but was important for acquisition of effector functions. Interestingly, IRF4 expression was unaltered in SEM4A4-deficient mice, yet effector differentiation was impaired, indicating that IRF4-dependent signaling may be necessary, but not sufficient, for differentiation of CD8^+^ effector T cells. Furthermore, the SEMA4A-plexin B2 axis shifts mTOR-mediated signaling from mTORC2 to mTORC1 in CD8^+^ T cells (63).

After expansion in the lymph nodes, effector CD8^+^ T cells travel to the site of infection to kill infected cells. Migration from the lymph node to the site of infection is in part, due to downregulation of CD62L and CCR7, which is dependent on activated mTOR (64). Such regulation by mTOR is evident from maintenance of CD62L and CCR7 in cells treated with rapamycin, leading to the accumulation of these cells in the secondary lymphoid organs rather than non-lymphoid tissue (65).

Following the dramatic CD8^+^ T cell expansion, and subsequent clearance of the pathogen, T cells go through a contraction phase leaving a resolute population of memory cells. The transition from effector to memory cells is coordinated with a transition from high mTOR activity to low mTOR activity. In contrast to CD8^+^ effector T cells, which favor glucose over fatty acid metabolism for energy, memory cells are less glycolytic and rely on lipid metabolism to break down fatty acids, amino acids, and glucose interchangeably (66).

The role of mTOR in regulating the transition from effector to memory cells has been studied extensively. Treatment of mice with a low dose of rapamycin or genetic depletion of *Raptor*-enhanced memory CD8^+^ T cell formation by promoting the generation of memory precursor cells, indicating that high mTOR signaling suppresses memory CD8^+^ T cell differentiation (67, 68). Similarly, adoptive transfer of LCMV-specific T cells cultured with rapamycin showed an increase in oxidative phosphorylation and were long-lived, compared to non-treated cells (69). Conversely, increasing mTOR via *Tsc1* deletion in T cells led to a decrease in memory T cells following *L. monocytogenes* infection, suggesting that TSC1 promotes memory CD8^+^ T cell formation by regulating mTOR activity (70). While reducing mTOR signaling increases memory T cells, it comes at the expense of an optimal primary effector response, which occurs due to impaired glycolysis in the CD8^+^ T cells after activation (59).

Although a low dose of rapamycin can augment memory formation in response to certain pathogens, long-term blockage of mTORC1 with the same low dose of rapamycin abrogated formation of memory CD8^+^ T cells in response to canary poxvirus vaccination (71). In this study, a short duration of a high dose of rapamycin during the expansion phase enhanced memory CD8^+^ T cell responses. Several factors could contribute to these observed differences in generating memory cells with rapamycin, including the strength of TCR stimulation, the level of mTOR activity induced with each pathogen, differential requirements and timing of pathogen replication, and experimental differences in the location (blood versus lymph node and spleen) where the T cells were analyzed. A recent study indicated that signaling through IFN-γR, with low, but not high TCR signaling, promoted mTOR signaling and generation of short lived effector cells, while simultaneously blocking the formation of memory precursors (72). Given that TCR signaling differs between pathogens, pathogen dose, and even between epitopes of the same pathogen (73), it is not surprising that rapamycin has varied impacts on the CD8 response to different pathogens. In addition, *Rictor*-deficient mice showed enhanced generation of memory CD8^+^ T cells, which implicates mTORC2 in suppressing memory cell formation (57). As mTORC2 does not impact formation of effector cells, yet suppresses memory formation, inhibition of mTORC2, rather than mTORC1, may provide an alternate means to boost CD8 memory T cells.

Studies investigating CD8-mediated tumor immunity demonstrate that mTOR controls memory CD8^+^ T cell differentiation by regulating two transcription factors, T-bet and Eomesodermin (Eomes) (74, 75). *In vitro* inhibition of mTOR with a low dose of rapamycin reduced T-bet expression and enhanced Eomes expression in CD8^+^ T cells, leading to augmented memory responses following adoptive transfer *in vivo* (74). A recent study indicates that mTOR also controls vaccinia virus-specific CD8^+^ T cell differentiation by regulating T-bet (76). Therefore, it is likely that mTOR similarly regulates memory differentiation in response to pathogens through T-bet and Eomes.

The effector to memory cell transition is also coupled with the cytokine environment, which appears to be critical for optimal memory development (77). Activation of mTOR by IL-7 promotes T-bet expression and upregulates IL-2Rβ. This, in turn, allows IL-15, which also utilizes IL-2Rβ for signaling, to promote differentiation of effector cells to memory cells through the upregulation of Eomes (75). IL-15 appears to be particularly important in promoting optimal memory following LCMV infection by transiently inducing cell-cycle progression, independent of antigen re-encounter, via a mTORC1-dependent pathway. This induces more rapid cell division and more protective memory cells following encounter with viral antigen (78). Thus, mTOR signaling impacts multiple stages of T cell activation and memory formation, and the impact of mTOR signaling can have different outcomes based on the stage of cellular differentiation.

An additional role for mTOR in regulating T cell exhaustion in response to chronic infection has emerged (79). T cell exhaustion refers to a state of dysfunction marked by reduced effector functions due to the persistence of antigen and inflammation (80). Following chronic LCMV infection, Akt and mTOR signaling were impaired in CD8^+^ effector T cells. This reduction in mTOR activity led to increased activity of the transcription factor, FOXO1 and subsequent upregulation of the inhibitory receptor, programed cell death protein 1 (PD-1), which promotes CD8^+^ T cell exhaustion (79). Blockade of PD-1 increased mTOR activity and decreased CD8^+^ T cell exhaustion. Furthermore, suppression of the mTOR pathway abrogated the therapeutic effects of PD-1 blockade, suggesting that mTOR activity is required to reverse T cell exhaustion by PD-1 blockade.

Recently, a population of long-lived, resident memory CD8^+^ T cells (T_RM_) that resides in peripheral tissues has been defined. T_RM_ cells are non-recirculating memory T cells located at barrier sites, including the skin and mucosal tissue (81, 82). Reactivation of T_RM_ stimulates innate immunity against antigenically unrelated pathogens, and could potentially enhance vaccine efficacy (83). While rapamycin promotes memory development in lymphoid tissues, it blocks formation of T_RM_ cells (84). Specifically, rapamycin treatment blocked formation of Vesicular stomatitis virus (VSV)-specific T_RM_ cells in the intestine and vaginal mucosa; however, lung T_RM_ cells were unaltered (84). Thus, mTOR appears to promote formation of mucosal resident memory cells in specific tissues following VSV infection. As such, promoting mTOR activation rather than inhibiting it may prove more beneficial in enhancing formation of T_RM_ cells.

In summary, signaling mediated by mTOR has diverse roles in CD8^+^ T cell-mediated immunity to pathogens including activation of naive precursors, migration of pathogen-specific cells, development of effector functions, regulation of chronically stimulated effector cells, and generation of stable memory cell populations. Each of these stages poses a potential for immune intervention. However, with such diverse roles, therapeutic regulation of the mTOR pathway will require extensive analysis of the potential implications.

## mTOR in CD4 T Cell Differentiation and Immunity to Pathogens

CD4^+^ T cells play both supportive and direct roles in host defense against pathogens. As helper cells they regulate the responses of innate immune cells, cytotoxic CD8^+^ T cells, and antibody-forming B cells. In particular, CD4 T cells are absolutely required for generating long-lived, protective antibodies. As direct effectors, CD4 T cells can utilize perforin-dependent cytolysis of infected cells (85, 86). Similar to CD8^+^ T cells, naive CD4^+^ T cells are initially primed in secondary lymphoid organs following binding of their TCR to microbial peptides presented by APCs. CD4^+^ TCR engagement, coupled with co-stimulation, leads to PI3K signaling, mTOR activation, and subsequent development into effector and memory populations (87).

Following antigen encounter, naive CD4^+^ T cells can differentiate into several distinct subsets including T helper cells (Th1, Th2, and Th17), T follicular helper cells (Tfh), effector cells, and regulatory T cells (Treg). Each CD4^+^ T cell lineage is associated with the expression of specific transcription factors: T-bet (Th1), Gata-3 (Th2), RORγt (Th17) Foxp3 (Treg), and Bcl-6 (Tfh) (88). mTOR is a key regulator of differentiation of CD4^+^ T cells. Specifically, differentiation into Th1, Th2, and Th17 cell lineages was severely inhibited in *mTOR*-deficient mice, even in the presence of polarizing cytokines (87, 89). In the absence of mTOR signaling, impaired phosphorylation of STATs in response to cytokine stimuli blocked the induction of the lineage specific transcription factors (89, 90). Conversely, suppression of mTOR with rapamycin or genetic deletion of *mTOR* in T cells promoted differentiation to FoxP3^+^ Treg cells, indicating that mTOR signaling blocks Treg development (89).

Subsequent studies using conditional deletion of *Raptor*, *Rheb*, or *Rictor* in T cells ascertained the contribution of mTORC1 and mTORC2 in T helper differentiation. Deficiency of *Rheb*, which is an activator of mTORC1 signaling, impaired differentiation of Th1 and Th17 cell lineages (91). However, *Raptor*-deficient T cells showed a defect in Th17, but not Th1 differentiation (92). More recently, it was shown that Th2 differentiation was also drastically compromised in the absence of *Raptor* (93). On the other hand, deficiency of *Rictor* and mTORC2 signaling impaired Th2 differentiation, but not Th1 or Th17 (91). However, a conflicting study suggested that mTORC2 signaling was also required for Th1 and Th2 differentiation (94). Furthermore, the loss of Th2 differentiation observed in *Rictor*-deficient T cells was comparatively minor compared to the loss observed following rapamycin treatment of *Rictor*-deficient T cells, indicating a more prominent role for mTORC1 in Th2 differentiation (93). Thus, it is clear that both mTORC1 and mTORC2 complexes play important roles in Th differentiation; however, the precise roles of each pathway in each of the subsets remain to be resolved.

Similar to CD8^+^ T cells, differentiation of naive CD4^+^ T cells to Th1, Th2, and Th17 effector cells represents a shift from oxidative phosphorylation to glycolysis. Th1, Th2, and Th17 cells express high levels of Glut1 and are highly glycolytic via mTOR signaling, which sustains their high energy consumption and supports their diverse effector functions (95). Development and maintenance of Th17 cells, in particular, is heavily reliant on glycolysis, which is stimulated by HIF-1α downstream of mTOR (96, 97). Therefore, signaling through mTOR contributes to resistance to a variety of pathogens via regulating transcription factors essential for developing T-helper subsets, and by maintaining glycolysis of these subsets following activation.

## mTOR in Treg Cell-Mediated Immunity to Infection

Regulatory T cells contribute most significantly to pathogen defense by suppressing T cell responses to limit immunopathology following infection (98). Foxp3^+^ expression distinguishes Treg cells from conventional T cells and confers Treg cell function (99, 100). Inhibition of mTOR signaling through either genetic deficiency or rapamycin treatment promoted expansion of preexisting Treg cells (89, 101–103) and induced the Treg cell phenotype on conventional T cells and Th17 cells (96, 104–106). Thus, mTOR is critical for negatively regulating Foxp3 expression and Treg cell numbers.

The stability and function of Treg cells is influenced by inflammation. In particular, Foxp3 Treg cells can be reprogramed into Th1 and Th17 effectors in the gut or sites of parasitic infection. However, reprograming of Treg cells into Th1/Th17 effectors is blocked with rapamycin, which stabilizes Foxp3 expression *in vivo* (107). Thus, mTOR signaling promotes differentiation of Treg cells into T helper cells when needed. Indeed, both Th17 and Treg cells require TGF-β for their differentiation, and the degree of mTOR activation delineates the relative development of each cell type. High levels of mTOR activation promote Th17 cell differentiation (108), whereas low mTOR signaling promotes Treg accumulation and sensitizes Treg cells to TGF-β (87, 109).

Furthermore, mTOR orchestrates a metabolic checkpoint for the differentiation between Treg cells and Th17 cells. Conditions that induced Th17 differentiation led to an induction of HIF-1α, which was dependent on mTOR signaling (96). HIF-1α, in turn, increased the expression of glycolytic enzymes and Th17 development, while simultaneously dampening Treg cell development. Thus, blocking glycolysis and mTOR promotes Treg cell generation by downregulation of HIF-1α (96). Similarly, the ratio between Th1 and Treg cells is also regulated by sphingosine 1-phosphate (S1P), which signals through mTOR and attenuates activity of SMAD3 to antagonize TGF-β, and inhibit generation of Treg cells and promote Th1 development (110).

In general, mTOR signaling suppresses Treg cell differentiation in favor of T helper differentiation, which increases immunity to pathogens by supporting the antimicrobial effector functions of T helper cells. Inhibition of mTOR can support development of Treg cells to restrain and control immune responses to circumvent excessive immunopathology. However, *Raptor* deficiency specifically in Treg cells unexpectedly impaired the suppressive function of Tregs and resulted in a fatal inflammatory disease, suggesting that the mTORC1 complex is also important to maintain Treg homeostasis and function (111). These seemingly contradictory data again highlight the complexity of the mTOR complexes, and the need to further delineate this signaling pathway.

## mTOR in Tfh Cell Immunity to Pathogens

Tfh cells are a subset of differentiated CD4^+^ T cells with a crucial role in initiating and maintaining germinal center reactions and high affinity isotype-class-switched antibody responses (112). Tfh cells express the transcription factor, Bcl6, which defines Tfh cells and promotes expression of the chemokine receptor, CXCR5 (113–115). In contrast to differentiation of Th1, Th2, and Th17 cells, which is supported by IL-2-mediated activation of mTOR, differentiation of Tfh cells is suppressed by IL-2 activation of mTOR. Th1 differentiation requires STAT5 activation and subsequent Blimp-1 expression, whereas Tfh cells develop when Blimp-1 synthesis is suppressed, enabling Bcl6 expression (116, 117). Indeed, Blimp-1 and Bcl6 expression are mutually exclusive, and over expression of either drives differentiation of Th1 or Tfh cells, respectively (113). During acute LCMV infection, Akt and mTOR signaling were essential for Blimp-1 and T-bet expression, which induced Th1 differentiation and countered Tfh development (118). Silencing of *Rictor* or *Raptor* demonstrated that mTORC1 suppresses Tfh cell development and induces Th1 cells, while mTORC2 may suppress Th1 cell development to permit preferential differentiation of Tfh cells in response to LCMV infection (118). Thus, commitment to either Th1 or Tfh lineages is discerned by the level of IL-2–STAT5–mTOR signaling, with increased signaling correlating with the Th1 transcription factors, T-bet and Blimp1, and lower IL-2–STAT5–mTOR levels with the Tfh transcription factor, Bcl6 (115, 116, 119–121).

A recent study demonstrated that during influenza virus infection, TGF-β opposes IL-2 to produce more Tfh cells and isotype-switched antibody responses rather than Th1 cells (122). TGF-β suppressed mTOR activation in T cells early during infection, which promoted Tfh cell differentiation by limiting IL-2 signaling. Inhibition of mTOR with rapamycin also promoted Tfh cell differentiation, suggesting that TGF-β restricts IL-2 responsiveness and insulates early Tfh progenitor cells from mTOR signaling to promote Tfh cell differentiation and isotype-switched antibody responses (122).

Bcl6 expression in Tfh cells downregulates genes associated with glycolysis, while T-bet in Th1 cells inhibits Bcl6-mediated repression of these genes to promote glycolysis (123). Accordingly, Tfh cells are less proliferative, and less glycolytic than Th1 cells, due to a lack of IL-2 signaling and a lower level of mTOR activation. Tfh cells therefore have a reduced metabolic capacity, similar to Treg cells, and also utilize oxidative phosphorylation for cellular maintenance (118).

Similar to the reciprocal relationship between Th17 and Treg cell activation, mTOR activation also plays a key role in regulating the balance between Th1 versus Tfh responses. While both Treg and Tfh cells are suppressed by mTOR activity and share metabolic similarities, Treg cells typically suppress and control immune responses, whereas Tfh cells typically promote immune responses, primarily germinal center formation and high affinity antibody responses.

## mTOR in B Cell-Mediated Immunity to Pathogens

B cells are responsible for producing pathogen-specific antibodies that block infection and control pathogen spread. Once generated, antibodies persist to provide long-lasting immunity. B cells develop in the bone marrow through several stages of maturation and differentiation that are influenced by mTOR signaling. Mice with a hypomorphic allele of mTOR have a partial block in B cell development in the bone marrow, and altered proportions of B cell subsets in the spleen (124). Conditional deletion of *mTOR* in B cells via CD19-cre did not abrogate development, but decreased mature, T2 transitional, and marginal zone B cells in the spleen (125). In addition, B cells lacking components of the mTORC2 complex had altered development. B cells deficient in *Sin1* accumulated at the pro-B cell stage in the bone marrow, and had a reduced capacity to become IgM^+^ immature B cells due to decreased Akt activation (126). Conditional deletion of *Rictor*, mediated by Vav-cre, did not perturb B cell development in the bone marrow, but did reduce mature B cells in the spleen (127). Interestingly, conditional deletion of *Tsc1* in B cells, which renders mTOR constitutively active, also impaired B cell maturation and significantly reduced marginal zone B cells (128, 129). Together, these studies show that both a reduction in mTOR activity and constitutive activation of mTOR can negatively impact B cell development, suggesting that the level of mTOR activation in B cells is critical for optimal B cell development.

Following development in the bone marrow, B cells migrate to secondary lymphoid organs and mature into follicular or marginal zone B cells, and remain quiescent until stimulated. B cells exhibit basal levels of mTOR activity in response to nutrients without stimulation. mTOR activity levels vary across B cell types, with marginal zone B cells maintaining high levels of mTOR activity, and follicular B cells having lower levels of mTOR activity (130). Activation through the BCR, TLR, or CD40 induces mTOR signaling (131). Similar to T cells, initial B cell activation increases glucose uptake and glycolysis to promote clonal expansion, which is also dependent on the mTOR pathway. Rapamycin inhibited B cell proliferation in response to anti-CD40, LPS, BAFF, and the polyclonal activator, *S. aureus* (131–136). In addition, B cells expressing the hypomorphic allele of mTOR had decreased proliferation in response to anti-IgM, anti-CD40, and LPS, with a bigger reduction following anti-IgM and anti-CD40 than LPS (124). These data demonstrate that mTOR signaling is essential for B cell proliferation in response to multiple stimuli.

Upon activation, B cells can either differentiate rapidly into plasmablasts that secrete low affinity IgM antibodies, or form germinal centers within secondary lymphoid organs, which are important for the generation of memory B cells and high affinity, isotype-class-switched antibody responses. As described above, the formation of germinal centers is dependent on interaction with Tfh cells. In the germinal centers, B cells proliferate and undergo somatic hypermutation (SHM) and class switch recombination (CSR). SHM increases antibody diversity by introducing point mutations into heavy chain Ig genes. CSR increases diversity further by rearranging the Ig heavy chain genes to express a constant region of a specific Ig antibody class. Initiation of these processes relies on activation-induced cytidine deaminase (*Aicda*; AID), which creates mutations in DNA by deaminating cytidine residues to generate uracil, leading to base pair mis-matching. Mutated B cells then undergo affinity-driven selection in the germinal centers, which is necessary for the generation of high affinity antibodies and optimal protection against pathogens.

Mechanistic target of rapamycin signaling is critical for the formation of germinal centers and the production of high affinity antibodies. Mice with a hypomorphic allele of mTOR had decreased antibody responses to T-independent and T-dependent antigens, with a more pronounced decrease in IgG compared to IgM antibodies (124). Further analysis of mice with the hypomorphic mTOR allele following immunization with either the model antigen NP-CGG, or heat-killed Pn14 derived from *S. pneumoniae* showed decreased germinal center formation, SHM, CSR, and IgG antibody affinity maturation (125). Challenge of mice with the hypomorphic mTOR allele with live *S. pneumoniae* led to reduced antibody titers and survival, compared to wild-type mice (125). Likewise, mice with a B cell-specific deletion of *mTOR* also had reduced germinal center formation and high affinity antibody generation following immunization with NP-CGG (125). In both cases, decreased mTOR signaling caused a reduction in the RNA and protein levels of AID. Rescue experiments, increasing AID levels in B cells with low mTOR activity, restored CSR, indicating that mTOR regulates the generation of high affinity antibodies through AID signaling (125).

Consistent with these findings, treatment of mice with a low dose of rapamycin during influenza vaccination blocked germinal center formation, *Aicda* transcription, and consequently, CSR (137). Furthermore, AID induction was dependent on signaling via mTORC1, as *Aicda* transcription was not induced in *Raptor*-deficient B cells following stimulation. In addition, mTOR was critical for CSR independent of proliferation, as the dose of rapamycin used in these experiments was low enough to support proliferation, yet *Aicda* transcription and CSR were inhibited (137).

Given that high affinity, class-switched antibodies are required for optimal protection against most pathogens, it was surprising that treatment of mice with a low dose of rapamycin during influenza vaccination improved protection against subsequent infections with different influenza subtypes (137). Analysis of the rapamycin-treated mice revealed a block in germinal center formation and CSR, which generated a broader antibody response, rather than a highly selected and affinity-matured repertoire. The majority of antibodies generated during influenza vaccination are high affinity antibodies specific for the globular head of the influenza hemagglutinin (HA) molecule. However, the HA protein is also the most variable protein between different influenza strains. Therefore, highly specific antibodies that protect against one subtype of influenza often do not protect against other subtypes. By blocking germinal center formation, rapamycin hampered the generation of high affinity antibodies specific for the variable region, with the unexpected benefit of allowing the lower affinity antibodies that are specific for conserved portions of influenza to become more prevalent. These data suggest that altering the levels of mTOR activation can steer the immune response away from strain-specific responses to more cross-reactive responses, which would be beneficial in generating a universal influenza vaccine.

It was also reported that mice with a conditional deletion of *Tsc1* in B cells, which renders mTOR constitutively active, also had a defect in germinal center formation, and a reduction in T-dependent and T-independent antibody production (128). These data suggest that the level of mTOR activation in B cells is critical for optimal germinal center formation. However, another group found that mice with a conditional deletion of *Tsc1* in B cells did not have defects in germinal center formation or high affinity antibody production (129). The reason for the discrepancy in these reports is not clear as both groups observed the same defects in B cell development in the absence of *Tsc1*.

Assessing the role of mTORC2 in antibody generation has also yielded seemingly conflicting data. In one study, inactivation of mTORC2, via *Rictor* deletion, impaired germinal center differentiation and antibody responses, demonstrating that mTORC2 supports antibody differentiation (127). However, in a subsequent study, partial mTORC2 inhibition through incomplete *Rictor* deletion increased CSR compared to wild-type mice, illustrating negative regulation of antibody responses by mTORC2 (138). Different levels of *Rictor* depletion or targeting B cells at different stages of development may have contributed to these discrepancies. The first study reported efficient deletion of *Rictor* in all hematopietic cells using Vav-cre, or a tamoxifen-inducible Cre (127). Whereas Limon and colleagues obtained partial *Rictor* depletion with CD19-cre (138). In addition, Limon et al. used varying doses of a new class of ATP-competitive mTOR kinase inhibitors to show that partial mTORC2 deletion enhances CSR. Partial deletion of mTORC1 or complete deletion of both mTORC1 and mTORC2 resulted in decreased, rather than increased CSR. However, partial inhibition of both mTOR complexes with inhibitors, or partial deletion of *Rictor* enhanced CSR. The increase in CSR was dependent on FOXO transcription factors (138). These data again highlight that the level of mTOR activation is a critical determinant on the cellular response.

Tfh cells and B cells act in unison to promote germinal center formation, CSR, and high affinity antibody responses. Yet, mTORC1 signaling typically suppresses Tfh cell development and function, while promoting CSR in B cells. While such a scenario does not create a quandary for cell-specific mTOR signaling, inhibition of the mTOR in the whole organism through either deletion of mTOR or with rapamycin treatment is likely to have opposing affects on antibody formation via Tfh cells and B cells. Similar to the self-regulating mechanism influencing differentiation of Treg cells versus Th1 cells or Tfh versus Th17 cells, the balance between B cell and Tfh cell function may be regulated by as yet unknown mechanisms. Initial inhibition of mTOR could allow development and function of Tfh cells to promote germinal center formation, followed by a period of enhanced mTOR activity to allow B cell proliferation and CSR to proceed.

## Conclusion

**Figure 2:**
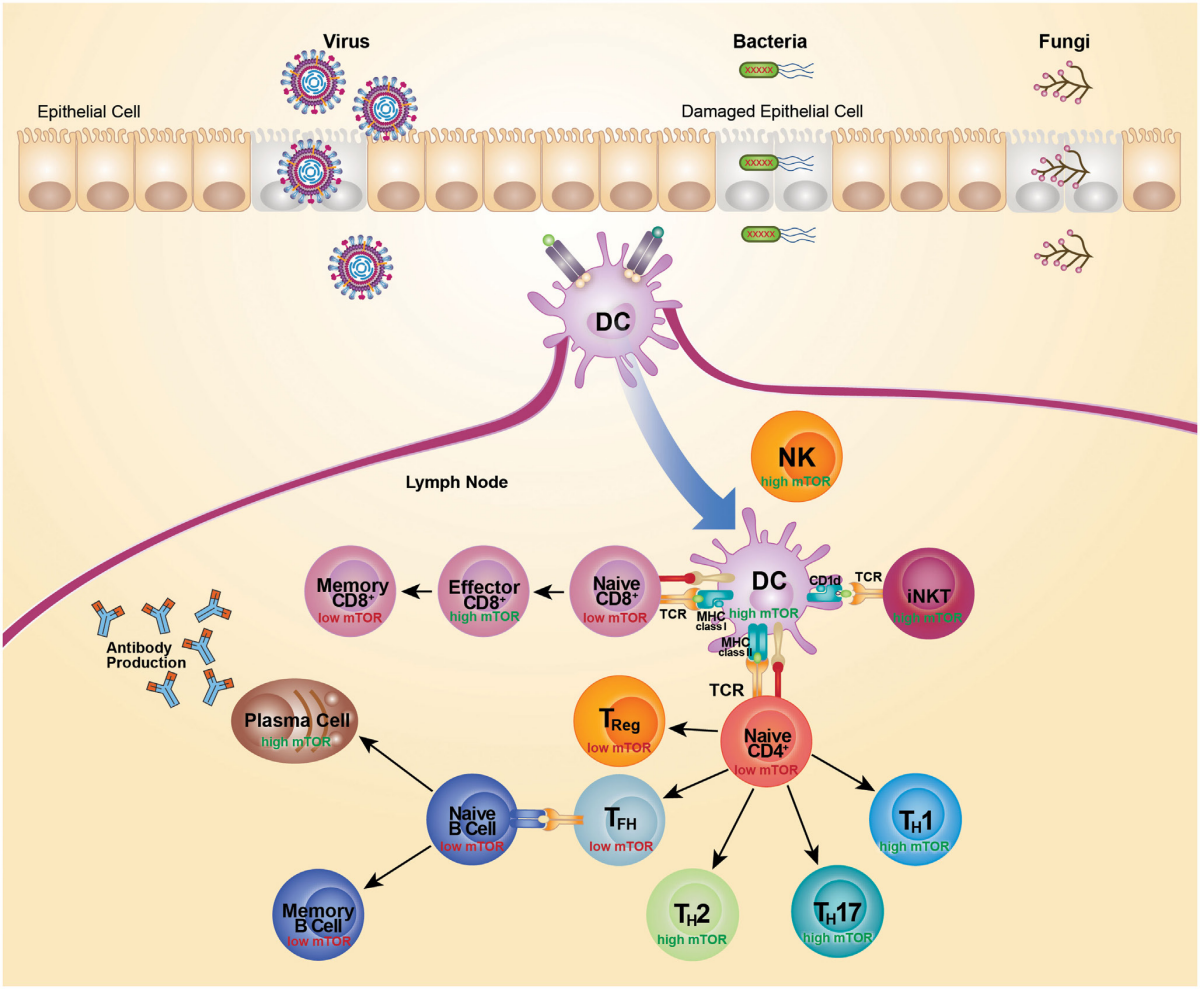
**mTOR activity differs across cell subsets**. Recognition of microbial components via TLRs leads to activation and subsequent migration of DC from the site of infection to the draining lymph nodes. Activated DCs upregulate costimulatory molecules to become highly efficient antigen-presenting cells. TCR recognition of microbial antigens presented by DC leads to activation and differentiation of CD4^+^ T cells, CD8^+^ T cells, and iNKT cells. This TCR-mediated activation is coupled with activation of mTOR and acquisition of effector functions. High mTOR activity is typically associated with high glycolytic and metabolic activity, whereas low mTOR activity is typically associated with low glycolytic activity and a more quiescent state. Activation of naive CD4^+^ T cells leads to an increase in mTOR activity and expression of transcription factors that promote the differentiation of Th1, Th2, and Th17 cells. Conversely, differentiation of CD4^+^ T cells in the absence of mTOR signaling promotes development of Treg cells and Tfh cells. Treg cell differentiation is supported by IL-2 and Tfh cell differentiation is suppressed by IL-2. Mature Tfh cells initiate germinal center reactions and support generation of high affinity isotype-class-switched antibody responses. High mTOR activity also supports the generation of CD8 effectors cells, whereas a dampening of mTOR activity supports the transition to memory CD8^+^ T cells. Cells are depicted in their activated state, unless specified as naive or memory.

It is now clear that mTOR has a central role in coordinating the outcome of pathogen defense by modulating immunity mediated by lymphoid cells. However, many details of the host–pathogen interaction, and their implications, are still to be determined. The mTOR-signaling pathway has a key role in regulating cell metabolism and is therefore readily hijacked by pathogens seeking to acquire energy and control cell death for their own propagation. Survival of a pathogen often necessitates that mTOR pathways are either activated or inactivated, largely to satisfy particular replication requirements. In turn, the counter immune response also varies depending on the type and dose of the infecting pathogen. Moreover, mTOR signaling varies across responding cell types, cell states, and response kinetics. As mTOR senses the immune microenvironment to direct cellular activation and differentiation, activity of mTOR parallels metabolic activity. Highly glycolytic cells exhibit the highest mTOR activity, while lower mTOR activity is associated with cells more reliant on oxidative phosphorylation for their energy needs (Figure 2). Use of metabolic inhibitors, such as rapamycin, that target the mTOR pathway would preferentially promote responses by more metabolically inactive Treg, Tfh, and memory cells, but would likely do so at the expense of Th1, Th2, Th17, CD8 effector, and antibody responses. As most cellular responses are intimately linked, opposing outcomes would need to be weighed against each other. The outcome of therapeutic intervention is also likely to be highly variable depending on the nature and stage of infection. Moreover, the precise level of mTOR activation is critical for particular immune responses as complete inhibition of a pathway can generate very different outcomes than partial inhibition. Selectively targeting components of the mTOR pathway and metabolic programing may prove more effective in designing vaccine strategies. For example, suppression of mTORC2 has potential for promoting the generation of memory CD8^+^ T cells, without negatively affecting the development of effector CD8^+^ T cells. The impact of any therapeutic approach on the metabolic pathway must be thoroughly anticipated and tested as it is clear that there is still much to learn about utilizing the mTOR pathway for successful therapeutic intervention.

## Author Contributions

RK and MM researched the literature and wrote the review.

## Conflict of Interest Statement

The authors declare that the research was conducted in the absence of any commercial or financial relationships that could be construed as a potential conflict of interest.
